# Nutrition items regulated by school nutrition policies in the USA and academic performance: a review of the non-policy literature

**DOI:** 10.1017/S1368980026102572

**Published:** 2026-04-30

**Authors:** Sydney Miller, Emma V. Sanchez-Vaznaugh, Megan Smith, Nancy Barba, Brisa Sánchez

**Affiliations:** 1 Department of Epidemiology and Biostatistics, Drexel University, USA; 2 Department of Public Health, San Francisco State University, USA

**Keywords:** Child health, Child nutrition, School interventions, Nutrition policies, Academic outcomes

## Abstract

**Objective:**

This systematic review synthesises existing research on the relationship between dietary intake – specifically concerning food and beverage items promoted or restricted by US federal school nutrition policies – and child academic performance, a salient predictor of long-term health.

**Design:**

We used keywords to search three databases. Along with other inclusion criteria, studies had to assess and report (i) a measure of intake of food groups/nutrient promoted or restricted by US school nutrition policies; (ii) a measure of academic performance and (iii) a measure of the association between both.

**Results:**

We identified thirty-nine studies, all of which utilised observational designs, and seven of which were considered higher quality based off the Quality Assessment Tool for Observational Cohort and Cross-sectional Studies, published by the National Heart, Lung, and Blood Institute. Thirty-five studies reported evidence that children had better academic outcomes when they had an increased intake of food groups and nutrient items promoted by school nutrition policies and/or a decreased intake of the food groups and nutrient items limited by school nutrition policies.

**Conclusion:**

These findings suggest that food groups and nutrient items governed by or promoted in school nutrition policies may shape children’s learning and contribute to downstream social determinants of health. Research on the population-level influences of school nutrition policies on children’s academic outcomes is warranted.

In the last twenty years, federal nutrition policies for schools in the USA and globally^(1)^ have been implemented to improve child nutrition. Given the considerable number of meals that children eat in schools, school nutrition policies are a viable avenue for improving childhood nutrition at the population level^(2)^. In the USA specifically, the historic Healthy, Hunger-Free Kids Act (HHFKA) was enacted to improve the affordability, accessibility and healthiness of school meals. Specifically, one critical component of this policy was that it aimed to better align school meals’ nutrition standards with national nutrition recommendations by increasing the availability and variety of fruits and vegetables and whole grains, limiting milk to fat-free or low-fat, reducing saturated and trans-fats and sodium (Na) and implementing age-based calorie limits^(3)^. In 2013, as part of HHFKA, ‘Smart Snacks for School’, also improved nutrition standards for snacks, food and beverages (also called ‘Competitive foods’) – sold to students in schools via vending machines, school stores, à la carte lines and in-school fundraisers^(4)^. Further, guidelines regarding HHFKA school meal policies were recently updated, with new provisions that began to go into effect in the 2025/26 school year (i.e. among other things, the policy progressively restricts the amount of added sugar in school meals)^(5)^.

Although school nutrition policies have well-established benefits for children’s dietary quality^(6–8)^, gaps remain in understanding whether the specific items and nutrients the policies promote or limit are associated with academic performance. Given evidence linking healthier diets to improved academic outcomes^(9–12)^, it is likely that school food policies influence academic performance through these targeted nutritional components. However, there are gaps in this body of literature. For example, policy-focused studies have examined the implementation of universal school meal programmes^(13)^ rather than how nutritional changes within school meals affect academic performance. Further, although considerable research has assessed individual-level diet quality and academic outcomes, systematic reviews are needed to incorporate the substantial body of evidence published since 2017^(11,12)^, as are reviews that directly consider the relevance to population-level nutrition interventions by focusing specifically on food and beverages regulated by federal school nutrition policies^(11,12)^. As such, there is no comprehensive, up-to-date review synthesising the evidence on how children’s academic outcomes are associated with intake of foods and nutrients promoted or restricted under federal school nutrition policies.

As federal policies to regulate food and beverages in schools are subject to revisions, for example, through reauthorisations, their relevance for child learning outcomes is of high significance. Yet, the lack of evidence in this area (both directly through policy research and indirectly through relevant literature reviews) limits the ability for this evidence to be considered in nutrition policy decision-making. The purpose of this review is to comprehensively synthesise whether children’s intake of specific items/nutrients regulated in the federal policies is associated with academic performance. This has important implications for the use of federal school nutrition polices to improve academic performance and downstream social factors such as educational achievement and socio-economic status that are fundamental determinants of health^(14–16)^.

## Methods

This review focuses on studies that assessed food groups targeted in federal school nutrition policies, as identified through our policy content analysis (Table 1), and their relationship with school-aged children’s academic performance, that includes standardised test scores, subject grades or grade point average (GPA) (Table 2). Additionally, food groups or nutrients were classified into whether they were promoted by the policies and thus are hypothesised to be positively associated with the academic outcomes (i.e. labelled as ‘nutrient-dense foods’), or if they were limited/restricted by the policies and are hypothesised to have negative effects on academic outcomes (i.e. labelled as ‘discretionary foods’ that are notably still present in school meals but are meant to be reduced in quantity or improved in quality).

**Table 1.** Categorisation of food groups and nutrients promoted or restricted in the school nutrition policies

Food group/drinks/nutrient	Policies
Nutrient-dense food groups to be increased	
Nonfat and low-fat milk	HHFKA 2012: All milk must be 1 % or non-fat
Fruits and vegetables	HHFKA 2012: Increased minimum number of servings of fruit and vegetables at both breakfast and lunch
Whole grains	HHFKA 2012: increased minimum number of servings of whole grains and introduced rolling implementation of requirement that 100 % of grains be whole grain; HHFKA 2018: relaxed whole-grain requirement to > 50 % whole grain, with remaining grains enriched; HHFKA 2022: increased whole-grain requirement to > 80 % whole grain, with remaining grains enriched
Discretionary food groups to be limited	
SSB	Smart snacks. Limitation on sugars in beverages and soda no longer allowed.
Nutrient-poor snack foods	Smart snacks. Limitation on nutrient-poor snack foods, by requiring that snacks’ first ingredient be one of the promoted food groups such as whole grains, fruits or vegetables and not exceed the limit on calories, fat, sugar and Na.
Fast foods^*^	Smart snacks. Nutrient standards for competitive food (a la carte) items that reduce availability of nutrient-poor items, by requiring that items’ first ingredient be one of the promoted food groups such as whole grains, fruits or vegetables and not exceed the limit on calories, fat, sugar and Na.
Sugar	HHFKA 2025. Rolling implementation of limits on sugar. Smart snacks. Limitation on percentage of weight from total sugar.
Calories	HHFKA 2012. Limitation of weekly average of calories served. Smart snacks. Limitation of maximum calories allowed per item.
Total fat, saturated fat and trans-fat	HHFKA 2012. Limitation on percentage of calories from saturated fat and limitation of zero grams trans-fat. Smart snacks. Limitation on percentage of calories from fat and saturated fat, < 0·5 grams trans-fat.
Na	HHFKA 2012. Rolling implementation of limits to total amount of Na served at lunch. HHFKA 2018 and 2022. Increases of final Na limits. HHFKA 2025. Decreases of final Na limits (however, final target is still higher than what was originally in HHFKA 2012). Smart snacks. Limitation of Na per item.

HHFKA, Healthy, Hunger-Free Kids Act; SSB, sugar-sweetened beverages.

* The policies do not explicitly name ‘fast food’; however, this label is used here to distinguish from snacks named in the previous row (including chips, candy, ice cream) and because most of the remaining competitive foods not already included in ‘snacks’ would be *a la carte* items such as pizza, burgers, etc., that are commonly referred to in the nutrition literature as fast food.

**Table 2.** Population/Problem, Intervention, Comparison, Outcomes, and Study Design (PICOS) criteria for research question

Participants	School-aged children
Intervention/exposure	Dietary intake promoted by federal school meal policies: higher intake of low-fat milk, fruits, vegetables and whole grains and lower intake of SSB, Na, sugar, snack foods, fast foods and fat
Comparison	Dietary intake that is less aligned with what is promoted by federal school meal policies
Outcomes	Academic outcomes
Study design	Observational (cross-sectional or longitudinal cohort studies), quasi-experimental or experimental

SSB – sugar-sweetened beverages.

The search protocol was registered online in Prospero as a systematic review (ID: CRD42023458882). Searches were completed using Pubmed, ERIC and SCOPUS and included studies up until 25 January 2026. The keywords used included school performance, academic achievement, academic performance, educational performance, scholastic performance, as well as terms broadly relevant to food and nutrient groups targeted by school nutrition policies: nutrition*, diet*, food, HEI, fat, whole grain, fiber, fruit*, vegetable*, sugar, sweet*, SSB, sugar-sweetened beverage*, milk, snack*, calori*, sodium, fast, fried, soda, ultra-processed. The terms ‘ultra-processed’ and ‘fast food’ do not explicitly appear in the language of the policies as directly regulated food items, but we include them in the search to be more comprehensive and because these foods are indirectly regulated through restrictions on nutrients such as sugar, Na, fat and calories. Moreover, the policies explicitly sought to improve competitive foods, including widely available a la carte items (e.g. pizza and burgers)^(17)^ that are traditionally labelled as fast food. Notably, these policies do not prohibit fast food items but aim to improve their healthiness through the nutrient-based restrictions, resulting in ‘fast food’ items that are lower in sugar, fat and Na^(5)^.

Inclusion criteria for targeted articles included: (1) the study population consisted of children and adolescents of school age, defined as 5–18 years old; and (2) at least one main aim of study was to examine how intake of specific nutrients, food and/or beverage items predicted academic outcomes. Additionally, (3) studies had to assess and report: (i) a measure of dietary intake that focused on food groups targeted by US school nutrition policies; (ii) a quantitative measure of academic performance recorded at the student level, such as GPA, school grades or reports, and standardised educational assessment test scores; and (iii) a measure of the association between dietary intake and academic performance; and (4) the study utilised a quantitative study design (observational or experimental). Though this review focuses on food groups targeted by US-based school nutrition policies, studies conducted in other countries were included, as we can expect the relationship between nutrition and academic performance to be generalisable across most groups of healthy children. The exclusion criteria for targeted articles were as follows: (1) studies on a different aspect of dietary behaviour, such as breakfast consumption, but not nutritional quality; (2) studies with children living in low-income countries, based on the World Bank definition^(18)^ (due to concerns with malnutrition, that is not as common or in the same fashion in US children); (3) qualitative studies; (4) non peer-reviewed publications, such as conference proceedings/abstracts; and (5) studies that focused on samples of children that had a specific disease or health condition. Additionally, (6) studies were excluded if the methods and results were unclear and key data such as effect measures could not be extracted.

All records that were retrieved by the initial search were imported into Covidence systematic review software (Veritas Health Innovation, https://www.covidence.org). Details on paper selection are illustrated in a PRISMA diagram (see online supplementary material, Supplemental Figure 1). All articles were screened, and data were extracted, independently by authors SM, MS and NB during the following four step process: (1) titles and abstracts were screened for inclusion; (2) full text was obtained for potentially eligible articles and assessed for inclusion; (3) data were extracted and quality assessment was completed and (4) discrepancies between data extractions and quality assessment were reconciled. Two authors completed each step, and disagreements were resolved via a third vote. Data extraction included the following: type of outcome measured (standardised tests, subject grades and GPA; and for which subjects and what the scoring mechanisms were), type of food and beverage assessed and how it was measured (i.e. use of FFQ, screener, etc.), study design (cross-sectional, longitudinal, quasi-experimental or experimental), the time frame of data collection, type of statistical analyses used and what variables were adjusted for, effect measures, key population characteristics, as well as whether analyses tested moderating factors and the results of those analyses.

To assess the quality of the papers, the three authors independently used the Quality Assessment Tool for Observational Cohort and Cross-sectional Studies, published by the National Heart, Lung, and Blood Institute^(19)^, given that the search did not identify any randomised controlled trials or experimental studies (see online supplementary material, Supplemental Figure 2). As part of quality assessment, we also considered the strength of the dietary measures used (i.e. the consensus by leading nutrition scientists is that FFQ and dietary screeners are considered weaker quality compared to multiple 24-h recalls or food diaries, though the vast majority of population-based studies use them^(20)^). This assessment tool is meant to be used flexibly, wherein reviewers use the quality assessment data to guide their decision in assigning a quality score to studies, although there is not a strict criterion for scores. Studies were assigned a score ranging from 1 to 3, with a 1 indicating there were major quality concerns, a 2 indicating the study had higher quality but suffered from methodological limitations (e.g. weaker dietary measure, cross-sectional analysis and less rigorous longitudinal study) and a score of 3 indicating the study had higher quality because of its methodological approach (e.g. robust longitudinal data, and/or a higher quality dietary assessment, see online supplementary material, Supplemental Figure 3).

## Results

We identified thirty-nine studies that met the criteria for inclusion (Table 3). The most common countries in which studies were conducted were the USA (*n* 11) and Canada (*n* 8). Studies looked at a variety of academic outcomes: subject grades (*n* 17), standardised tests (*n* 16) and overall GPA (*n* 8). The most common academic subjects that were assessed were Math (*n* 18) and English and Language Arts (ELA, *n* 21), and many studies assessed multiple different academic subjects (e.g. standardised test scores for Math, ELA and other subjects, all as separate outcomes).

**Table 3.** Characteristics of studies included in review of dietary intake and children’s academic outcomes

Citation	Country	*N*	Age or grade (baseline)	Dietary assessment^*^	Academic outcome	Quality rating^†^
Highest quality (rating of 3)						
Burrows, 2017^(29)^	Australia	4245	Age 8–15 (years)	Screener	Standardised test	3
Dubuc *et al.*, 2019^(30)^	Canada	187	Grade 7–9	Screener	GPA	3
Faught, 2019^(31)^	Canada	11 016	Grades 9–12	Screener	Subject grade	3
Li and O’Connell, 2012^(32)^	USA	6178	Kind	Screener	Standardised test	3
Purtell and Gershoff, 2015^(33)^	USA	8544	Grade 5	Screener	Standardised test	3
Smith and Richards, 2018^(34)^	UK	1660	Grades 7–11	Screener	Subject grades	3
Tobin, 2013^(35)^	USA	12 000	Grade 5	Screener	Standardised test	3
Quality rating of 1 or 2						
Aquilani *et al.*, 2011^(21)^	Italy	40	Avg. age 15 years	Food diary (multiple)	Subject grades	1
Asigbee *et al.*, 2018^(46)^	USA	9720	Grade 8	Screener	Standardized test	2
Barchitta *et al.*, 2019^(58)^	Italy	213	Age 15–18 years	Screener	Subject grades	1
Bleiweiss-Sande *et al.*, 2019^(10)^	USA	868	Age 8–10 years	Screener	Standardised test	1
Burns, 2018^(44)^	USA	4625	Grades 9–12	Screener	GPA	1
Correa-Burrows, 2015^(52)^	Chile	1073	Age 13–15 years	Screener	Standardized test	2
Correa-Burrows, 2016^(53)^	Chile	395	Age 16 years	Screener	GPA; standardised test	2
Correa-Burrows, 2017^(51)^	Chile	678	Age 16–17 years	Screener	GPA	2
Crispin & Ruiz, 2025^(49)^	Mexico	189	Age 14–16 years	Screener/Plate Analysis	GPA	2
Edwards *et al.*, 2011^(47)^	USA	800	Grade 6	Screener	Standardised test	1
Faught, Gleddie *et al.*, 2017^(43)^	Canada	28 608	Age 11–15 years	Screener	Subject grades	2
Faught, Ekwaru *et al.*, 2017^(48)^	Canada	4253	Grade 5	Screener	Standardised test	2
Faught, Montemurro *et al.*, 2017^(36)^	Canada	1595	Grade 5	Screener	Standardised test	2
Florence *et al.*, 2008^(54)^	Canada	5200	Grade 5	Screener	Standardised test	2
Kim *et al.*, 2003^(39)^	Korea	6463	Grades 5, 8 and 11	Screener	GPA	1
Kim *et al.*, 2016^(42)^	Korea	359 264	Age 12–18 years	Screener	Subject grades	2
Littlecott *et al.*, 2016^‡^^(23)^	Wales	1217	Grades 5–6 years	Screener	Standardised test	2
Lopez-Gil *et al.* ^(59)^	Spain	788	Ages 12–17 years	Screener	GPA, subject grades	2
Maniaci *et al.*, 2023^(55)^	Italy	373	Avg. age 17 years	Screener	GPA	2
Martínez-Gómez *et al.*, 2012^(37)^	Spain	1825	Age 13–17 years	Screener	Subject grades	2
McIsaac *et al.*, 2015^(50)^	Canada	535	Grades 4–6	Screener	Subject grades	2
Nigg and Amato, 2015^‡^^(26)^	USA	334	Grades 4–6	Screener	Subject grades	2
Nyaradi *et al.*, 2015^(56)^	Australia	779	Age 14 years	Screener	Standardised test	2
Papasideris, 2021^(27)^	Canada	67	Age 13–18 years	Screener	Subject grades	1
Pearce, 2018^(57)^	Australia	315	Age 9–11 years	Screener	Standardised test	2
Ptomey *et al.*, 2016^(24)^	USA	698	Grade 2–3	Breakfast recall	Standardised test	1
Qin *et al.*, 2024^(28)^	China	217	Age 12–17 years	Screener	Subject grades	2
Rasberry *et al.*, 2017^(41)^	USA	15 624	Grades 9–12	Screener	Subject grades	2
Ren *et al.*, 2022^(25)^	China	9251	Grade 7	Screener	Subject grades	2
Sigfusdotti *et al.*, 2007^(45)^	Iceland	5810	Age 14–15 years	Screener	Subject grades	2
Snelling *et al.*, 2015^(40)^	USA	1000	Grades 6–12	Screener	Subject grades	1
Stea & Torstveit, 2014^(38)^	Norway	2432	Age 15–17 years	Screener	Subject grades	2

GPA, grade point average.

Authors’ analysis of data from studies extracted for review.

* Screener includes FFQ or other validated dietary questionnaire.

† Highest score is a 3, indicating highest level of quality.

‡ Study uses data from longitudinal study to conduct cross-sectional analyses.

### Assessment of study quality

Studies varied in methodological rigour, including study design, dietary assessment methods and quality assessment (Table 3, and see online supplementary material, Supplemental Figure 3). Seven studies were assigned a higher quality rating of 3 due to notable methodological strengths such as the use of a rigorous analysis of longitudinal data or the use of quasi-experimental designs. These studies are hereafter referred to as ‘higher quality’, but not gold standard since they did not use true experimental designs, and none used the highest quality dietary assessment methodology^(20)^. Twenty-three studies were given a quality rating of 2, meaning they had general methodological limitations (i.e. cross-sectional data, weak though adequate dietary assessment^(20)^). Nine studies were given a quality rating of 1, because they had additional quality concerns, despite two of these studies using the strongest dietary assessment methods of multiple dietary recalls^(21,22)^. The most common quality concerns for these nine studies were lack of adjustment for confounders, lack of or unclear variable definition and having less than a 50 % participation rate.

### Study findings

Tables 4, 5 and 6 summarise study findings on food items and nutrients’ relationships with academic outcomes. We have grouped findings by three categories: (1) nutrient-dense food groups or items, (2) discretionary food groups or items and (3) intake of multiple food groups or items addressed by school nutrition policies (see online supplementary material, Supplemental Figure 4 for details about specific food groups and nutrient items included in each study). Because some patterns of eating are expected to be associated with better academic outcomes, and some with worse outcomes, we group the description of findings as (1) supportive findings, wherein findings were as expected: (a) a higher intake of a nutrient-dense item and/or food group that is promoted by school nutrition guidelines was associated with better academic outcomes, or (b) a higher intake of a discretionary item and/or food group that was limited by school nutrition guidelines was associated with worse academic outcomes; and (2) non-supportive or null findings, wherein the findings were the opposite direction as expected, or the item and/or food group was not significantly associated with academic outcomes.

**Table 4.** Association between food groups (regulated by US school nutrition policies) with children’s academic outcomes

	Nutrient-dense food groups promoted by the policies		Discretionary food groups/nutrients limited or restricted by the policies					
	Fruits and vegetables	Whole grain	SSB	Sugar	Snack foods	Fast food	Calorie	Fat
**Overall GPA or subject grades**								
Overall GPA								
Burns, 2018	Veg↑							
Corea-Burrows, 2017					↓			
Crispin & Ruiz, 2025	–							
Dubuc *et al.*, 2019^‡^	↑*							
Faught, Gleddie *et al.*, 2017	↑				↓			
Kim *et al.*, 2003							↓	
Kim *et al.*, 2016	↑		↓			–		
Nigg and Amato, 2015	↓							
Rasberry *et al.*, 2017	Fruit↑		↓					
Snelling *et al.*, 2015	Veg↑		↓			↓		
Subject grades – combined grades of select subjects								
Faught, Montemurro *et al.*, 2017	–	–	↓*	↓^*,§^				–^||^
Littlecott *et al.*, 2016	↑				↑			
Stea & Torstveit, 2014	Fruit↑ Veg↑^‡^		↓		↓^‡^			
Kim, Kim & Kang, 2016								
Ren *et al.*, 2022			↓				↑	
Sigfusdotti *et al.*, 2007	↑							
**Math**								
Standardised tests								
Asigbee *et al.*, 2018	↑							
Bleiweiss-Sande *et al.*, 2019	–				–			
Burrows, 2017^‡^	Fruit↑		–			–		
Correa-Burrows, 2015					↓			
Edwards *et al.*, 2011	–		↓					
Faught, Ekwaru *et al.*, 2017	–	–		–^§^				–^||^
Li and O’Connell, 2012^‡^			–		–	↓		
Ptomey *et al.*, 2016	–	↑	Juice ↓	–^§^				–
Purtell and Gershoff, 2015^‡^						↓		
Tobin, 2013						↓		
Subject grade								
Faught *et al.*, 2019^‡^	↑	–						
Kim, Kim & Kang, 2016								
Martínez-Gómez *et al.*, 2012	Fruit ↑^‡^							
McIsaac *et al.*, 2015			–					
Papasideris, 2021						–		
**ELA**								
Standardised tests								
Asigbee *et al.*, 2018	↑							
Bleiweiss-Sande *et al.*, 2019	Fruit↓				↓^3^			
Burrows, 2017^‡^	Veg↑		↓			–		
Correa-Burrows, 2015					↓			
Edwards *et al.*, 2011	–		↓					
Faught, Ekwaru *et al.*, 2017	–	↑		↓^§^				↓^||^
Li and O’Connell, 2012^‡^			–		–	↓		
Ptomey *et al.*, 2016	–	↑	Juice ↓	–^§^				–
Purtell and Gershoff, 2015^‡^						↓		
Tobin, 2013						↓		
Subject grade								
Faught *et al.*, 2019 ^‡^	↑	–						
Kim, Kim & Kang, 2016								
Martínez-Gómez *et al.*, 2012	Fruit ↑ ^‡^							
McIsaac *et al.*, 2015			↓					
Papasideris, 2021						–		
**Other subjects**								
Writing (all standardised tests)								
Burrows, 2017^‡^	↑		↓			–		
Faught, Ekwaru *et al.*, 2017	–	–		↓^§^				–^||^
Nyaradi *et al.*, 2015								
Spelling (standardised test)								
Ptomey *et al.*, 2016	–	–	–	–^§^				–
Science (standardised test)								
Asigbee *et al.*, 2018	↑							
Purtell and Gershoff, 2015^‡^						↓		

GPA, grade point average; ELA, English and Language Arts.

Authors’ analysis of data from studies extracted for review.

↑ associated with higher academic marks; ↓ associated with lower academic marks; -- not significantly associated with academic marks; blank cell means not examined in the study.

* Boys only.

† Girls only.

‡ Higher quality rating.

§ Added sugar.

|| Saturated fat.

**Table 5.** Association between intake of multiple food groups regulated by US school nutrition policies with children’s academic outcomes

	Overall aligned diet	Higher intake of multiple nutrient-dense foods	Higher intake of multiple discretionary foods
**Overall GPA or subject grades**			
Overall GPA			
Correa-Burrows, 2016	↑		
Lopez-Gil *et al.* ^(59)^			↓
Maniaci *et al.*, 2023		↑	
Subject grades – combined grades of select subjects			
Barchitta *et al.*, 2019			–
Sigfusdotti *et al.*, 2007			↓
Smith and Richards, 2018^‡^			–
**Math**			
Standardised tests			
Asigbee *et al.*, 2018			–
Bleiweiss-Sande *et al.*, 2019			↓
Correa-Burrows, 2016	↑		
Nyaradi *et al.*, 2015		–	↓
Pearce, 2018			↓
Subject grade			
Aquilani *et al.*, 2011		↑^§^	
Barchitta *et al.*, 2019			–
Lopez-Gil *et al.* ^(59)^			↓
McIsaac *et al.*, 2015	–		
Qin *et al.*, 2024	–		
**ELA**			
Standardised tests			
Asigbee *et al.*, 2018			–
Bleiweiss-Sande *et al.*, 2019			↓
Correa-Burrows, 2016	↑		
Florence *et al.*, 2008	↑		
Nyaradi *et al.*, 2015		–	↓
Pearce, 2018			↓
Subject grade			
Barchitta *et al.*, 2019			↓
Lopez-Gil *et al.* ^(59)^			↓
McIsaac *et al.*, 2015	↑		
Qin *et al.*, 2024	–		
**Other subjects**			
Writing (standardised tests)			
Nyaradi *et al.*, 2015		–	–
Italian (subject grades)			
Barchitta *et al.*, 2019			↓
Science (standardised tests)			
Asigbee *et al.*, 2018			–

GPA, grade point average; ELA, English and Language Arts.

Authors’ analysis of data from studies extracted for review.

↑ associated with higher academic marks; ↓ associated with lower academic marks; -- not significantly associated with academic marks.

*Overall aligned diet reflects a diet that is higher in nutrient-dense foods like fruits, vegetables and whole grains and lower in discretionary foods like sugar, Na, fast food, etc. Intake of nutrient-dense foods reflects a diet high in nutrient-dense foods (without considering intake of discretionary foods), and intake of discretionary foods reflects a diet high in discretionary food groups (without considering intake of nutrient-dense foods).

‡ Males only.

§ Females only.

**Table 6.** Number of supportive findings^*^ by subject and quality rating

	Math				ELA				Other/All			
	Rating of 1 or 2^†^		Rating of 3^†^		Rating of 1 or 2^†^		Rating of 3^†^		Rating of 1 or 2^†^		Rating of 3^†^	
	*n*	%	*n*	%	*n*	%	*n*	%	*n*	%	*n*	%
Nutrient-dense food groups												
Fruit and vegetables	2	33 %	2	100 %	2	33 %	2	100 %	9	70 %	2	100 %
Whole grains	1	50 %	0	0/1^‡^	2	100 %	0	0/1^‡^	0	0/2^‡^	0	0/1^‡^
Discretionary food groups												
SSB	2	67 %	0	0/2^‡^	3	100 %	1	50 %	7	89 %	1	100 %
Snack foods	1	50 %	0	0/1^‡^	2	100 %	0	0/1^‡^	3	75 %	N/A	
Sugar	0	0/2^‡^	N/A		1	50 %	N/A		2	67 %	N/A	
Fast food	1	50 %	2	67 %	1	50 %	2	67 %	1	50 %	1	50 %
Calories	N/A		N/A		N/A		N/A		1	50 %	N/A	
Fat	0	0/2^‡^	N/A		1	50 %	N/A		0	0/3^‡^	N/A	
Intake of multiple food groups												
Overall aligned diet	1	33 %	N/A		3	75 %	N/A		1	100 %	N/A	
Nutrient-dense foods	1	50 %	N/A		0	0/1^‡^	N/A		1	50 %	N/A	
Discretionary foods	4	67 %	N/A		5	83 %	N/A		3	60 %	0	0/1^‡^

ELA, English and Language Arts.

Authors’ analysis of data from studies extracted for review.

* Supportive findings: The findings of the study show that dietary intake that better aligns with intake regulated by school nutrition policies are associated with better academic outcomes.

† Numbers are the number of each type of study that had supportive findings, also expressed as a percentage of the total *n* of that type of study.

‡ Numerator indicates zero studies had supportive findings, and denominator is total *n* of relevant studies.

Of the thirty-nine studies included in this review, thirty-one studies reported evidence that that *at least one* dietary characteristic promoted/regulated by federal school nutrition policies was statistically significantly linked with better academic outcomes (Tables 4 and 5). Additionally, four of the thirty-nine studies reported mixed findings^(10,23–25)^. Lastly, one study found that dietary characteristics were linked with only worse outcomes^(26)^, and three studies had only null findings^(27,28)^. Along with Tables 4 and 5, in the sections below, we describe the studies’ results. Due to the complexity of describing effect sizes of studies with various dietary predictors and academic outcomes, we provide more details on the specific effect sizes only from those studies that were given a rating of 3 (*n* 7)^(29–35)^. We describe studies with a quality rating of 1 or 2 more generally, unless findings differed for girls *v*. boys, as existing evidence suggests gender differences in consumption^(21,36–39)^. We also note if there were only studies available for limited age ranges (e.g. if there were four studies on one food group, and all four studies were conducted with elementary students). Though most studies found at least one of the dietary characteristics promoted by nutrition guidelines were linked with better outcomes, findings were variable by food/nutrient groups, academic outcome and study quality (Table 6).

### Nutrient-dense food groups or items promoted by school nutrition policies

#### Milk

None of the studies we identified in the review examined consumption of low fat or non-fat milk, as regulated by the policies. Our review identified nine studies that examined milk consumption without specifications as to fat content, as described in the discussion.

#### Fruits and vegetables

A total of eighteen studies examined fruits and vegetables, with thirteen finding supportive associations^(23,29–31,37,38,40–46)^, five finding null associations^(10,24,47–49)^ and two2 findings unsupportive associations^(10,26)^.

##### Supportive findings

Thirteen studies found that a higher intake of fruit and/or vegetables was significantly associated with better academic outcomes, with ten of these studies having a quality rating of 1 or 2, and three of these studies having a higher quality rating of 3^(23,29–31,37,38,40–46)^. The first higher quality study found that high school students who consumed at least 7–8 servings of fruits and vegetables per day had 1·23 times the odds of achieving a higher grade in Math (95 % CI 1·01, 1·49), and 1·33 times the odds of achieving a higher grade in ELA (95 % CI 1·11, 1·60)^(31)^. The second higher quality study found that, among children aged 8–15 years, higher vegetable consumption was associated with higher standardised test scores, with the greatest effect on writing (mean difference of 86·0 ± 26·5 points between children who consumed vegetables 7 evenings per week *v*. 0 evenings/week, *P* < 0·01)^(29)^. Additionally, children who ate fourteen pieces of fruit per week had higher writing scores than children who consumed 3–4 pieces per week (mean difference of 17·0 ± 5·7 points, *P* = 0·04). The third higher quality study found that among seventh to ninth grade students, an increase in daily servings of fruit and vegetables over a period of three years was linked with higher GPA at follow-up, but only for boys (b = 0·5, *P* = 0·000)^(30)^.

Additionally, two of the other studies found different results for girls *v*. boys: one study with a quality rating of 1 found that fruits were beneficial for Math and ELA outcomes for girls only^(37)^, while another study with a quality rating of 2 found that fruits were beneficial for both boys’ and girls’ overall GPA, but vegetables were beneficial for girls only^(38)^.

##### Unsupportive or null findings

Four studies with a quality rating of 2 found no significant associations between fruit and vegetable intake and academic outcomes^(24,47–49)^.

Additionally, two studies with a quality rating of 2 reported significant negative associations, with higher fruit and vegetable intake being associated with worse outcomes^(10,26)^.

#### Whole grains

A total of four studies examined whole grains, with two finding supportive associations^(24,48)^ and two finding null associations^(31,36)^.

##### Supportive findings

Two studies with a quality rating of 2 used samples of elementary schoolchildren and found that a higher intake of whole grains was significantly associated with better academic outcomes^(24,48)^.

##### Unsupportive or null findings

Two studies found that whole grain intake was not significantly associated with academic outcomes, with one of these studies having a quality rating of 2 and one having a higher quality rating of 3^(31,36)^. The higher quality study found no significant associations with subject grades in Math or ELA among a sample of high school students in Canada^(31)^.

### Discretionary food groups or items regulated or limited by school nutrition policies

#### Sugar-sweetened beverages

A total of eleven studies examined whole grains, with nine finding supportive associations^(24,25,29,36,38,40–42,47)^ and two finding null associations^(32,50)^.

##### Supportive findings

Nine studies found that a higher intake of sugar-sweetened beverages (SSB) was significantly associated with worse academic outcomes, with eight of these studies having a quality rating of 1 or 2, and one of these studies having a higher quality rating of 3^(24,25,29,36,38,40–42,47)^. The higher quality study found that among children aged 8–15 years, higher SSB consumption was significantly associated with lower standardised test scores, with the greatest effect being observed in reading scores (mean difference of 46·1 ± 12·9 points between children who drank 4–6 glasses/d *v*. < 1 glass/d, *P* < 0·01)^(29)^.

Additionally, one of the studies with a quality rating of 2 found different results for girls *v*. boys, where SSB were linked with significantly worse combined subject grades in Math and ELA among boys only^(36)^.

##### Unsupportive or null findings

Two studies found that SSB intake was not significantly associated with academic outcomes, with one study having a quality rating of 2 and one having a rating of 3^(32,50)^. The higher quality study found that among elementary school students in the USA, SSB intake was not significantly associated with standardised test scores in Math or ELA^(32)^.

#### Sugar

A total of three studies examined sugar, and all three found supportive associations^(10,36,48)^. All three studies focused on elementary school students and focused specifically on added sugar rather than total sugar.

##### Supportive findings

Three studies with a quality rating of 2 found that increased intake of sugar was significantly associated with worse academic outcomes^(10,36,48)^. One of these studies found that increased intake of sugar was significantly associated with lower combined subject grades in Math and ELA for boys only^(36)^.

##### Unsupportive or null findings

None.

#### Nutrient-poor snack foods

Studies examined snack foods that were calorie-dense or nutrient-poor. A total of seven studies examined snack foods, with four finding supportive associations^(38,43,51,52)^, two finding null associations^(10,32)^ and one finding unsupportive associations^(23)^.

##### Supportive findings

Four studies with a quality rating of 2 found that a higher intake of snack foods was significantly associated with worse academic outcomes among elementary and high school students^(38,43,51,52)^. One of these studies found that snack foods were linked with worse outcomes in combined subject grades for girls only^(38)^.

##### Unsupportive or null findings

Two studies found that snack foods were not significantly associated with academic outcomes, with one of them having a quality rating of 2 and one of them having a higher quality rating of 3^(10,32)^. The higher quality study found that among elementary school students, intake of snack foods was not significantly associated with standardised test scores in Math or ELA^(32)^.

Lastly, a study with a rating of 2 unexpectedly found that intake of snack foods was significantly associated with improved academic outcomes among middle school students^(23)^.

#### Fast food

A total of seven studies examined fast foods, with four finding supportive associations^(32,33,35,40)^ and three finding null associations^(27,29,42)^.

##### Supportive findings

Four studies found that increased intake of fast food was significantly associated with worse academic outcomes, with 1 having a quality rating of 2 and 3 of them having a higher quality rating of 3^(32,33,35,40)^. The first higher quality study found that children who consumed fast food daily gained significantly fewer points from fifth to eighth grade than children who consumed no fast food, in the subjects of reading (b = –4·10, *P* < 0·01), math (b = –3·22, *P* < 0·001) and science (–3·46, *P* < 0·01); and there were also some negative effects for children consuming fast food multiple times per week^(33)^.

The second higher quality study found that, among fifth graders, as frequency of fast food consumption increased, standardised test scores in Math decreased by 2·60 points and growth rates were 0·03 points lower per month^(32)^. For reading, as frequency of fast food increased, standardised test scores decreased by 2·87 points and reading growth rates were 0·04 points lower per month^(32)^. Finally, the last higher-quality study found that fifth graders who consumed fast food 4 to 6 times per week had significantly lower test scores in both reading and math^(35)^.

##### Unsupportive or null findings

Three studies found that fast food intake was not significantly associated with academic outcomes, with one having a quality rating of 1, one having a rating of 2 and one having a higher quality rating of 3^(27,29,42)^. The higher-quality study focused on children aged 8–15 years in Australia and found that fast food intake was not associated with Math or ELA standardised test scores^(29)^.

#### Calories

A total of two studies examined calories, with one finding supportive associations^(39)^ and one finding unsupportive associations^(25)^.

##### Supportive findings

One study with a quality rating of 2 found that increased energy intake was significantly associated with worse academic outcomes^(39)^.

##### Unsupportive or null findings

One study with a quality rating of 1 found unsupportive findings. Among middle school students, moderate energy intake was associated with higher subject grades than low energy intake^(25)^.

#### Fat

A total of three studies examined dietary fat, with one finding supportive associations^(36)^ and two finding null associations^(24,48)^. All three studies focused on elementary school students.

##### Supportive findings

One study with a quality rating of 2 found that higher saturated fat intake was associated with worse academic outcomes^(36)^.

##### Unsupportive or null findings

Two studies with a quality rating of 2 found that saturated fat^(48)^ and total fat^(24)^ were unrelated to academic outcomes.

#### Sodium

Na content is regulated by school nutrition policies, but we identified no studies examining Na.

### Intake of multiple food groups or items addressed by school nutrition policies

#### Overall aligned diet

An ‘overall aligned diet’ was defined as a diet that included a higher intake of multiple nutrient-dense foods promoted by US school nutrition policies and a lower intake of multiple discretionary foods limited by US school nutrition policies (e.g. higher intake of fruits and vegetables and whole grains, and lower intake of sugar and fast food). A total of four studies examined an overall aligned diet, with three finding supportive associations^(50,53,54)^ and one finding null associations^(28)^.

##### Supportive findings

Three studies with a quality rating of 2 found that an overall aligned diet had a significant positive association with academic outcomes^(50,53,54)^.

##### Unsupportive or null findings

One study with a quality rating of 2 found that an overall aligned diet was not significantly associated with academic outcomes^(28)^.

#### Intake of multiple nutrient-dense foods

There were studies that examined intake of multiple nutrient-dense foods promoted by US school nutrition policies (e.g. intake of fruits, vegetables and whole grains, etc.). A total of three studies examined intake of multiple nutrient-dense foods, with two finding supportive associations and one finding null associations.

##### Supportive findings

Two studies with a quality rating of 2 found that higher intake of nutrient-dense foods was associated with significantly better academic outcomes^(21,55)^.

Additionally, one of these studies found improved subject grades in Math among girls only^(21)^.

##### Unsupportive or null findings

One study with a quality rating of 2 found that intake of nutrient-dense foods was not associated with academic outcomes^(56)^.

#### Intake of multiple discretionary foods

Some studies examined intake of multiple discretionary foods regulated by US school nutrition policies (e.g. intake of SSB and fast food, etc.). A total of eight studies examined intake of multiple discretionary foods, with six finding supportive associations^(10,45,56–59)^ and two finding null associations^(34,46)^.

##### Supportive findings

Six studies with a quality rating of 1 or 2 found that higher intake of discretionary foods was significantly associated with worse academic outcomes^(10,45,56–59)^.

##### Unsupportive or null findings

Two studies found that intake of discretionary foods was not significantly associated with academic outcomes, with one of these studies having a quality rating of 2 and one having a higher quality rating of 3^(34,46)^. The higher-quality study found that intake of discretionary foods was not associated with combined subject grades in Math and ELA among children in grades 7–11 in the UK^(34)^.

## Discussion

The overwhelming majority of studies found that the dietary intake promoted and regulated by US school nutrition policies were significantly associated with improved academic performance outcomes among children. Although the studies we reviewed were observational and had general methodological limitations, the findings as a whole provide evidence to support that increased intake of one, or a combination of, nutrient-dense food groups promoted by HHFKA (i.e. fruits and vegetables, whole grains) is associated with better academic performance. Similarly, there is evidence to support that decreased intake of one, or a combination of, discretionary foods limited by HHFKA and competitive foods policies (i.e. SSB, fast food, nutrient-poor snack foods and sugar) is linked with worse academic performance.

This review bridges a gap in the literature by synthesising the evidence on the associations between specific food and beverage items targeted by school nutrition policies and student academic performance. This synthesis of the evidence lends further support to the argument that there is an intermediary pathway between school nutrition policies and academic performance^(60–62)^; thus, school nutrition policies have important implications not only for immediate health outcomes but also for academic outcomes. This has critical implications for downstream health consequences, as academic performance is strongly associated with adult educational attainment and socio-economic status, a salient social determinant of health^(14–16)^. Future policies and programmes should continue to aim to increase consumption of fruits and vegetables and whole grains and decrease the consumption of SSB, foods high in sugars, fat, salt and nutrient-poor snack foods in schools, particularly in schools serving socially disadvantaged students.

Evidence from studies with higher quality identified in this review was most robust for increasing intake of fruits and vegetables (e.g. three higher quality studies that supported this, as well as thirteen more other studies) and decreasing intake of fast food (e.g. three higher quality studies, as well as one more study that supported this). There was also sufficient evidence to support limiting intake of SSB (e.g. nine supportive studies, one of which was higher quality). The evidence was limited for whole grains, fat and energy intake, given few studies were available on these specific food groups. There was also slightly stronger evidence for overall GPA and performance in English Language Arts, as virtually all studies that examined these outcomes had supportive findings. Additionally, of the five studies that looked at gender differences, three found evidence among females only and two that found evidence among males only. Notably, both studies that found evidence among males only were based on Canadian populations. Otherwise, there were no discernable differences in findings across different countries.

In addition to the studies included in this review, we identified several relevant studies examining dietary patterns and academic outcomes. Seven met our inclusion criteria but did not provide extractable data; their findings were generally consistent with included studies^(63–69)^. One study found that carotenoids – an objective marker of fruit and vegetable intake – were associated with better academic outcomes, but it was excluded because this review focused on self-reported intake^(70)^. We also identified studies on dietary factors not directly targeted by US school nutrition policies. Of nine studies on milk consumption,^(10,22,24,31,36,41,42,47,48)^ six reported positive associations with academic outcomes^(22,31,36,41,42,48)^, though these were excluded because milk fat levels were unspecified. Studies of the Mediterranean diet^(71–79)^, as well as higher consumption of nuts^(80)^, fish^(81,82)^, micronutrients^(83)^ or meat^(31,48)^, also found positive associations. Collectively, this evidence suggests that additional nutrients and food groups may warrant consideration in school nutrition policies to support academic performance.

### Limitations and future directions

All studies included in this review were observational, as we identified no randomised controlled or experimental studies through our search. Further, most studies used lower-quality dietary assessment methods such as screeners, although these methods have the advantage of collecting population-based data and thus enhance generalisability. Future research is needed to include higher-quality studies that make use of both longitudinal and experimental data along with stronger dietary measures, such as multiple 24-h recalls or food diaries. However, this synthesis of the available evidence provide support for the conclusion that food, nutrients and beverages that are promoted in the US school nutrition policies was associated with better academic performance.

Future research directions include exploring some of the food and nutrient groups that were understudied in the body of evidence we reviewed here, such as whole grains, energetic intake and saturated fat consumption. Quasi-experimental studies that explicitly examine the impact of HHFKA implementation on academic outcomes are needed to expand our understanding of the possible influences of these policies on multiple child outcomes.

### Conclusion

Modifications for federal nutrition policies for schools are considered every five years during the process of the Child Nutrition Reauthorization^(3)^. In recent years, HHFKA has been amended to reduce some of its standards, and there is ongoing debate on whether to keep or reverse these rollbacks. This review suggests that these school nutrition policies have the potential to promote child health and learning. As low-income and non-White children see the greatest dietary improvements as a result of school nutrition policies^(84–87)^, these policies may also reduce dietary disparities and lessen the ‘academic achievement gap’ that exists for these underserved populations^(60–62)^. Given that children’s exposure to foods at school is widespread, enforcing and strengthening HHFKA and competitive foods policies is a promising population-level intervention to interrupt the trajectory from the academic achievement gap to downstream disparities in SDOH (Social Determinants of Health) and related health outcomes^(88–91)^. However, it is important to note that schools that cater to underserved children also tend to have the most barriers to complying with school nutrition guidelines; thus, additional financial and logistical support for these schools is needed^(92)^.

## Supporting information

10.1017/S1368980026102572.sm001 Miller et al. supplementary material Miller et al. supplementary material

## Supplementary material

For supplementary material accompanying this paper visit https://doi.org/10.1017/S1368980026102572.
