# Supplementary material for: Nutrition items regulated by school nutrition policies in the USA and academic performance: a review of the non-policy literature
Source: Public Health Nutr. 2026 Apr 30;29(1):e119. doi: 10.1017/S1368980026102572 (PMC13434496; doi:10.1017/S1368980026102572)
Supplement: Supplementary file 1 [file S1368980026102572sup001.docx]

Supplemental Figure 1. PRISMA guidelines for study selection.

| Supplemental Figure 2. Quality Assessment Tool  Adapted from the Quality Assessment Tool for Observational Cohort and Cross-sectional Studies, published by the National Heart, Lung, and Blood Institute^(1)^ | |
| --- | --- |
| Q1 | Was the research question or objective in this paper clearly stated? |
| Q2 | Was the study population clearly specified and defined? |
| Q3 | Was the participation rate of eligible persons at least 50%? |
| Q4 | Were all the subjects selected or recruited from the same or similar populations (including the same time period)? |
| Q5 | Were inclusion and exclusion criteria for being in the study prespecified and applied uniformly to all participants? |
| Q6 | Was a sample size justification, power description, or variance and effect estimates provided? |
| Q7 | For the analyses in this paper, were the exposure(s) of interest measured prior to the outcome(s) being measured? |
| Q8 | Was the timeframe sufficient so that one could reasonably expect to see an association between exposure and outcome if it existed? |
| Q9 | Were the exposure measures (dietary variables) clearly defined? |
| Q10 | Is the dietary measure used the strongest possible measure (i.e., multiple 24 hour recalls, rather than FFQ, dietary screener, or other method)? |
| Q11 | Was the exposure(s) assessed more than once over time? |
| Q12 | Were the outcome measures (academic variables) clearly defined, valid, reliable, and implemented consistently across all study participants? |
| Q13 | Was loss to follow-up after baseline 20% or less? |
| Q14 | Were key potential confounding variables measured and adjusted statistically for their impact on the relationship between exposure(s) and outcome(s)? |

| **Supplemental Figure 3. Quality assessment results for studies included in review.** Y=Yes, study meets the criteria; N=No, study does not meet criteria, or study is not clear | | | | | | | | | | | | | | | |
| --- | --- | --- | --- | --- | --- | --- | --- | --- | --- | --- | --- | --- | --- | --- | --- |
|  | **Q1** | **Q2** | **Q3** | **Q4** | **Q5** | **Q6** | **Q7** | **Q8** | **Q9** | **Q10** | **Q11** | **Q12** | **Q13** | **Q14** | **Rating** |
| *Aquilani et al., 2011^(2)^ | Y | Y | N | Y | Y | N | Y | Y | Y | Y | Y | Y | N | N | 1 |
| Asigbee et al., 2018^(3)^ | Y | Y | Y | Y | Y | Y | N | Y^1^ | Y | N | N | Y | N/A | Y | 2 |
| Barchitta et al., 2019^(4)^ | Y | Y | N | Y | Y | Y | N | Y^1^ | Y | N | N | Y | N/A | N | 1 |
| Bleiweiss-Sande et al., 2019^(5)^ | Y | Y | N | Y | Y | Y | N | Y^1^ | Y | N | N | Y | N/A | Y | 1 |
| Burns, 2018^(6)^ | Y | Y | Y | Y | Y | Y | N | Y^1^ | Y | N | N | Y | N/A | Y | 1 |
| *Burrows, 2017^(7)^ | Y | Y | N | Y | Y | Y | Y | Y | Y | N | Y | Y | N | Y | 3 |
| Correa-Burrows, 2015^(8)^ | Y | Y | Y | Y | Y | Y | N | Y^1^ | Y | N | N | Y | N/A | Y | 2 |
| Correa-Burrows, 2016^(9)^ | Y | Y | Y | Y | Y | Y | N | Y^1^ | Y | N | N | Y | N/A | Y | 2 |
| Correa-Burrows, 2017^(10)^ | Y | Y | N | Y | Y | Y | N | Y^1^ | Y | N | N | Y | N/A | Y | 2 |
| Crispin & Ruiz, 2025^(11)^ | Y | Y | Y | Y | Y | Y | N | Y^1^ | Y | N | N | Y | N/A | Y | 2 |
| *Dubuc et al., 2019^(12)^ | Y | Y | N | Y | Y | Y | Y | Y | Y | N | Y | Y | Y | Y | 3 |
| Edwards et al., 2011^(13)^ | Y | Y | N | Y | Y | Y | N | Y^1^ | Y | N | N | Y | N/A | N | 1 |
| Faught, 2017a^(14)^ | Y | Y | Y | Y | Y | Y | N | Y^1^ | Y | N | N | Y | N/A | Y | 2 |
| Faught, 2017b^(15)^ | Y | Y | Y | Y | Y | Y | N | Y^1^ | Y | N | N | Y | N/A | Y | 2 |
| Faught, 2017c^(16)^ | Y | Y | N | Y | Y | Y | N | Y^1^ | Y | N | N | Y | N/A | Y | 2 |
| *Faught, 2019^(17)^ | Y | Y | Y | Y | Y | Y | Y | Y | Y | N | Y | Y | N | Y | 3 |
| Florence et al., 2008^(18)^ | Y | Y | Y | Y | Y | Y | N | Y^1^ | Y | N | N | Y | N/A | Y | 2 |
| Kim et al., 2003^(19)^ | Y | Y | Y | Y | Y | Y | N | Y^1^ | Y | N | N | Y | N/A | N | 1 |
| Kim et al., 2016^(20)^ | Y | Y | Y | Y | Y | Y | N | Y^1^ | Y | N | N | Y | N/A | Y | 2 |
| *Li and O'Connell, 2012^(21)^ | Y | Y | N | Y | Y | Y | N | Y | Y | N | N | Y | Y | Y | 3 |
| Littlecott et al., 2016 ^(22)^ | Y | Y | Y | Y | Y | Y | Y | Y | Y | N | Y | N | N/A | Y | 2 |
| Lopez-Gil et al.^(23)^ | Y | Y | Y | Y | Y | Y | N | Y^1^ | Y | N | N | Y | N/A | Y | 2 |
| Maniaci et al., 2023^(24)^ | Y | Y | Y | Y | Y | Y | N | Y^1^ | Y | N | N | Y | N/A | Y | 2 |
| Martínez-Gómez et al., 2012^(25)^ | Y | Y | Y | Y | Y | Y | N | Y^1^ | Y | N | N | Y | N/A | Y | 2 |
| McIsaac et al., 2015^(26)^ | Y | Y | N | Y | Y | Y | N | Y^1^ | Y | N | N | Y | N/A | Y | 2 |
| *Nigg and Amato, 2015^(27)^ | Y | Y | N | Y | Y | Y | Y | Y | Y | N | Y | Y | N | Y | 2 |
| *Nyaradi et al., 2015^(28)^ | Y | Y | Y | Y | Y | Y | N | Y^1^ | Y | N | N | Y | N | Y | 2 |
| Papasideris, 2021^(29)^ | Y | Y | N | Y | Y | N | N | Y^1^ | Y | N | N | Y | N/A | N | 1 |
| Pearce, 2018^(30)^ | Y | Y | Y | Y | Y | Y | N | Y^1^ | Y | N | N | Y | N/A | Y | 2 |
| Ptomey et al., 2016^(31)^ | Y | Y | Y | Y | Y | N | N | N | Y | N | N | Y | N/A | N | 1 |
| *Purtell and Gershoff, 2015^(32)^ | Y | Y | Y | Y | Y | Y | Y | Y | Y | N | N | Y | Y | Y | 3 |
| Qin et al., 2024^(33)^ | Y | Y | Y | Y | Y | Y | N | Y^1^ | Y | N | N | Y | N/A | Y | 2 |
| Rasberry et al., 2017^(34)^ | Y | Y | Y | Y | Y | Y | N | Y^1^ | Y | N | N | Y | N/A | Y | 2 |
| Ren et al., 2022^(35)^ | Y | Y | Y | Y | Y | Y | N | Y^1^ | Y | N | N | Y | N/A | Y | 2 |
| Sigfusdotti et al., 2007^(36)^ | Y | Y | Y | Y | Y | Y | N | Y^1^ | Y | N | N | Y | N/A | Y | 2 |
| *Smith and Richards, 2018^(37)^ | Y | Y | Y | Y | Y | Y | Y | Y | Y | N | Y | Y | N | Y | 3 |
| Snelling et al., 2015^(38)^ | Y | Y | Y | Y | Y | N | N | Y^1^ | Y | N | N | Y | N/A | N | 1 |
| Stea & Torstveit, 2014^(39)^ | Y | Y | Y | Y | Y | Y | N | Y^1^ | Y | N | N | Y | N/A | Y | 2 |
| Tobin, 2013^(40)^ | Y | Y | Y | Y | Y | Y | N | Y^1^ | Y | N | N | Y | N/A | Y | 3 |

*Study collects longitudinal data

Q8: Y^1^ – study uses a dietary screener that is not administered prior to outcome, but assesses dietary behavior over a time period preceding the outcome

Q13 was N/A for cross-sectional studies

| **Supplemental Figure 4. Foods Included in Studies Jointly Evaluating Multiple Food Groups Summarized as an Index or Score** | |
| --- | --- |
| Asigbee et al., 2018^(3)^ | Discretionary foods: Fast foods, snack foods, SSBs |
| Barchitta et al., 2019^(4)^ | Discretionary foods: Fast foods, snack foods, sugar |
| Bleiweiss-Sande et al., 2019^(5)^ | Discretionary foods: Snack foods, SSBs |
| Correa-Burrows, 2016^(9)^ | Overall aligned diet: Better score indicates higher intake of fruits and vegetables, and lower intake of fat, sugar, sodium, calories |
| Florence et al., 2008^(18)^ | Overall aligned diet: Diet Quality Index (better score indicates higher intake of fruits, vegetables, and whole grains, and lower intake of snack foods and fast foods) |
| Lopez-Gil et al.^(23)^ | Discretionary foods: Fast foods, snack foods, SSBs |
| Maniaci et al., 2023^(24)^ | Nutrient-dense foods: Whole grains, fruits, and vegetables |
| McIsaac et al., 2015^(26)^ | Overall aligned diet: Diet Quality Index (better score indicates higher intake of fruits, vegetables, and whole grains, and lower intake of snack foods and fast foods) |
| Nyaradi et al., 2015^(28)^ | Discretionary foods: Fast foods, snack foods, SSBs |
| Pearce, 2018^(30)^ | Discretionary foods: Fast foods, snack foods, SSBs |
| Sigfusdotti et al., 2007^(36)^ | Discretionary foods: Fast foods, snack foods |
| Smith and Richards, 2018^(37)^ | Discretionary foods: Fast foods, snack foods |
